# Compensatory Upregulation of LPA_2_ and Activation of the PI3K-Akt Pathway Prevent LPA_5_-Dependent Loss of Intestinal Epithelial Cells in Intestinal Organoids

**DOI:** 10.3390/cells11142243

**Published:** 2022-07-20

**Authors:** Zhongxing Liang, C. Chris Yun

**Affiliations:** 1 Division of Digestive Diseases, Department of Medicine, Emory University School of Medicine, Atlanta, GA 30322, USA; zliang@emory.edu; 2 Gastroenterology Research, Atlanta Veterans Administration Medical Center, Decatur, GA 30322, USA; 3 Winship Cancer Institute, Emory University School of Medicine, Atlanta, GA 30322, USA

**Keywords:** lysophosphatidic acid, LPA5, intestine, epithelial cells, organoid

## Abstract

Renewal of the intestinal epithelium is orchestrated by regenerative epithelial proliferation within crypts. Recent studies have shown that lysophosphatidic acid (LPA) can maintain intestinal epithelial renewal in vitro and conditional deletion of *Lpar5* (*Lpar5^iKO^*) in mice ablates the intestinal epithelium and increases morbidity. In contrast, constitutive *Lpar5* deletion (*Lpar5^cKO^*) does not cause a defect in intestinal crypt regeneration. In this study, we investigated whether another LPA receptor (LPAR) compensates for constitutive loss of LPA_5_ function to allow regeneration of intestinal epithelium. In *Lpar5^cKO^* intestinal epithelial cells (IECs), *Lpar2* was upregulated and blocking LPA_2_ function reduced proliferation and increased apoptosis of *Lpar5^cKO^* IECs. Similar to *Lpar5^cKO^* mice, the absence of *Lpar2* (*Lpar2^−/−^*) resulted in upregulation of *Lpar5* in IECs, indicating that LPA_2_ and LPA_5_ reciprocally compensate for the loss of each other. Blocking LPA_2_ in *Lpar5^cKO^* enteroids reduced phosphorylation of Akt, indicating that LPA_2_ maintains the growth of *Lpar5^cKO^* enteroids through activation of the PI3K-Akt pathway. The present study provides evidence that loss of an LPAR can be compensated by another LPAR. This ability to compensate needs to be considered in studies aimed to define receptor functions or test the efficacy of a LPAR-targeting drug using genetically engineered animal models.

## 1. Introduction

The surface of the intestine is lined with a continuous monolayer composed of a variety of epithelial cells, each having specific roles to maintain the critical function of the intestine in nutrient absorption and fluid secretion. In addition, the intestinal epithelium provides a chemical and physical barrier that protects the host from the toxic luminal environment. The intestinal epithelium renews every 3–5 days through a process involving regeneration of new cells by a stable population of intestinal stem cells (ISCs), marked by the leucine-rich repeat-containing G protein-couple receptor 5 (LGR5), residing at the bottom of the crypt [1]. The ISCs give rise to daughter cells that continue to divide and differentiate as they move up towards the surface of the lumen and eventually slough off before being replaced by newly generated cells. ISCs in the crypt base are maintained by multiple signaling molecules secreted by the surrounding stromal environment that regulate the Wnt/b-catenin, Notch, bone morphogenic protein (BMP), epidermal growth factor (EGF), and Hedgehog pathways [2]. In 2009, Sato and co-workers developed a 3-D culture system that indefinitely maintains intestinal stem cells as organoids that form epithelial structures mimicking the crypt–villus structure of the small intestine in a culture dish [3].

In the dynamic environment of the intestine, growth factors have been established to be important mediators of cellular proliferation, differentiation, migration, and survival [4,5]. Lysophosphatidic acid (LPA) is a naturally occurring phospholipid that mediates growth factor-like effects on various cell types and tissues [6]. The effects of LPA are mediated through six distinct LPA receptors (LPARs), termed LPA_1_-LPA_6_ (gene names *Lpar1*-*Lpar6*), which activate major cellular signaling pathways, including the Wnt/b-catenin, phosphatidylinositol-3-kinase (PI3K), and mitogen-activated protein kinase (MAPK) pathways [6]. Each LPAR is coupled to at least one or more of the heterotrimeric Gα proteins, including Gα_i/o_, Gα_q/11_, Gα_12/13_, and Gα_s_, and LPAR expression varies widely among different tissues and cell types. This variation in LPA receptor expression in part accounts for the dichotomy of LPA-mediated effects observed in different tissues and cell types [7]. The signaling of LPA through LPARs induces diverse effects that are potential mediators during pathological conditions, including rheumatoid arthritis, pulmonary fibrosis, neurodegenerative diseases, and cancer, making LPARs and LPA-mediated signaling potential therapeutic targets. Indeed, research and development of molecules targeting LPARs and the pathways associated with LPA-mediate signaling are underway [8].

To date, knockout (KO) mice have been reported for all six known receptors, and studies of these mice have revealed new pathophysiological roles of LPAR. A targeted deletion of *Lpar1* results in neonate lethality with impaired sucking. Surviving *Lpar1*-deficient (*Lpar1^−/−^*) mice exhibit abnormal phenotypes, such as craniofacial dysmorphism with shorter snouts, wider-spaced eyes, and reduced brain mass [9]. Mice lacking *Lpar2* are grossly normal, but an increased incidence of perinatal frontal hematoma is observed in *Lpar1*/*Lpar2* double KO (*Lpar1^−/−^/Lpar2^−/−^*) mice compared with *Lpar1^−/−^* mice, indicating a modest role of LPA_2_ in vascular development [9,10]. *Lpar3*-deficiency results in delayed implantation and embryo crowding and, as a result, *Lpar3^−/−^* female mice produce reduced litter sizes [11]. *Lpar4*-deficiency results in defective blood and lymphatic vessel formation during mouse embryogenesis leading to neonatal deaths [12]. A recent study has shown the synergistic role of LPA_4_ and LPA_6_ in vascular development and that *Lpar4*/*Lpar6* double KO embryos die due to vascular deficiencies with enlarged aortae and poor vascular networks [13].

Mice constitutively lacking *Lpar5*, *Lpar5^−/−^*, grossly appear normal although decreased pain sensitivity and anxiolytic phenotype are present [14,15]. These mice have a defective intestinal epithelial barrier, which renders them more sensitive to chemical-induced colitis, but otherwise have a normally functioning gastrointestinal system [16]. In contrast, conditional KO of *Lpar5* in adult mice results in acute inflammation in the intestinal tract which increases morbidity and mortality of the animals [17]. Loss of *Lpar5* in ISCs decreases stem cell functions and reduces the expansion of progenitors [17]. The absence of a gross phenotype by constitutive *Lpar5* KO suggests that LPA_5_ functions are masked by yet unknown compensatory responses. Here, using IEC-derived enteroids, we show that LPA_2_ compensates for the absence of LPA_5_ and activates the PI3K-Akt pathway, promoting IEC proliferation and survival.

## 2. Materials and Methods

### 2.1. Mice

The generation of *Lpar5^f/f^* and *Lpar5^f/f^; Villin-Cre* mice was previously described [18]. *Lpar5^f/f^; RosaCre^ERT^* and *Lpar5^f/f^; AhCre* mice were recently described [17].

### 2.2. Intestinal Crypt Isolation and 3-D Culture of Enteroids

Mouse small intestinal crypts were cultured as previously described [3]. Briefly, the isolated small intestine was cut longitudinally and washed with cold PBS. Crypts were incubated for 1 h at room temperature in Gentle Cell Dissociation Reagent (Stemcell Technologies, Vancouver, BC, Canada) and released from tissue by gentle agitation. Crypts were then passed through a 70 μm cell strainer and the crypt fraction was enriched by centrifugation. Crypts were embedded in Matrigel and cultured in growth media (50% Advanced DMEM/F-12, 50% L-WRN, 10% FBS, N-2 media supplement, B-27 supplement, 100 units/mL penicillin, and 0.1 mg/mL streptomycin). Media were replenished every 2–3 days.

### 2.3. Treatment of Enteroids with Inhibitors

*Lpar5^f/f^;Rosa-Cre^ERT^* and *Lpar5^f/f^;Ah-Cre* enteroids were treated with 1 μM 4-hydroxytamoxifen (4OHT) or 1 µM β-naphthoflavone (β-NF), respectively. *Lpar5^f/f^* and *Lpar5^f/f^;Villin-Cre* enteroids were treated with an equal volume of sunflower oil. Where indicated, enteroids were treated with 10 μM Ki16425, 10 μM H2L51186303 (Tocris Bioscience, Minneapolis, MN, USA), 10 μM AS2717638 (Fisher Scientific, Hampton, NH, USA), 50 μM LY294002, or 1 μM AG1478 (Sigma-Aldrich, St. Louis, MO, USA). After the formation of enteroids, the media containing inhibitors or carrier were added to enteroids for 48 h.

### 2.4. Treatment of Mice with LPA_2_ Antagonist

*Lpar5^f/f^* and *Lpar5^f/f^;Villin-Cre* mice, sex- and age-matched, were given LPA_2_ antagonist H2L5186303, 10 mg/kg body weight, by intraperitoneal injection every other day for three times. On day 7, each mouse was administered 5-ethynyl-2′-deoxyuridine (EdU) and euthanized 2 h later.

### 2.5. Immunofluorescence Staining of Mouse Intestine

After flushing with cold PBS, small intestinal tissues were incubated overnight in 30% sucrose in PBS for cryoprotection. Six mm-thick cryostat sections were prepared and stored at −80 °C until needed. The frozen sections were fixed with ice-cold 100% ethanol and acetone at the ratio of 1:1 for 10 min at −20 °C. EdU-labeled cells were stained using the Click-iT EdU Cell Proliferation kit (Thermo Fisher). Terminal deoxynucleotide transferase dUTP nick end labeling (TUNEL) staining was performed on intestinal tissues and enteroids using an in situ cell death detection kit (MilliporeSigma, St. Louis, MO, USA) according to the manufacturer’s instruction. Images were taken using a Nikon A1R HD confocal microscope (Nikon Instruments, Melville, NY, USA).

### 2.6. Immunofluorescence Staining of Enteroids

Enteroids cultured in eight-well chamber slides (Nunc Laboratory-Tek II, MilliporeSigma, Burlington, MA, USA) were fixed with 4% paraformaldehyde for 10 min at room temperature. After fixation, enteroids were permeabilized with 0.2% Triton X-100 for 10 min and blocked in PBS containing 5% goat serum for 1 h at room temperature prior to incubation overnight with rabbit anti-mouse pan-Akt antibodies (4691, Cell Signaling) or rabbit anti-mouse phospho-Akt antibodies (4060, Cell Signaling) at 1:400 dilutions at 4 °C. Slides were then washed with PBS and incubated with Alexa fluor 568 conjugated goat anti-rabbit secondary antibodies (A11036, Invitrogen) at a 1:500 dilution for 45 min at room temperature. After washing with PBS, enteroid cells were counterstained with Hoechst (Thermo Fisher Scientific, Waltham, MA, USA) for nuclei. Finally, the slides were mounted with Prolong gold anti-fade reagent (Thermo Fisher Scientific). The slides were viewed under a Nikon A1R HD25 confocal microscope system with 40× oil objective lens, and images were acquired with the NIS-Elements C Imaging software (Nikon Instruments, Melville, NY, USA).

### 2.7. Quantitative RT-PCR (qRT-PCR)

Enteroids were harvested to extract total RNA with the RNeasy Mini kit (Qiagen, Hilden, Germany). One μg of total RNA was used for cDNA synthesis using the First Strand cDNA Synthesis Kit (Thermo Fisher Scientific) according to the manufacturer’s instruction. Quantitative PCR was performed with iQ SYBR Green Supermix (Bio-Rad Laboratories, Hercules, CA, USA) on the Mastercycler Realplex (Eppendorf, Hamburg, Germany). PCR primer sequences are listed in Table 1.

### 2.8. Statistical Analysis

Statistical analysis was performed by unpaired Student’s *t*-test, one-way or two-way ANOVA, followed by Tukey post hoc analysis using GraphPad Prism software (Version 9.4, GraphPad Software, San Diego, CA, USA). Results are presented as mean ± SD. A value of *p* < 0.05 was considered significant.

## 3. Results

**Figure 1:**
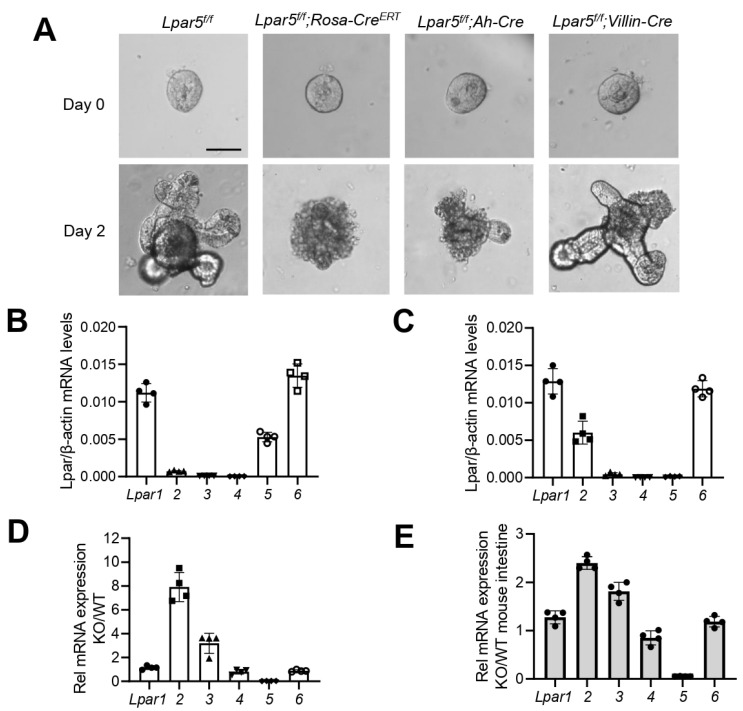
Constitutive deletion of *Lpar5* is associated with growth of enteroids and upregulation of *Lpar2 and Lpar3*. (**A**) Enteroids were derived from control *Lpar5^f/f^*, mice with inducible deletion of *Lpar5* (*Lpar5^f/f^;Rosa-Cre^ERT^*, *Lpar5^f/f^;Ah-Cre*), and mice with constitutive, intestinal-specific deletion of *Lpar5* (*Lpar5^f/f^;Villi-Cre*). *Lpar5^f/f^;Rosa-Cre^ERT^* and *Lpar5^f/f^;Ah-Cre* were treated with 4OHT or β-NF, respectively, on Day 0 (D0) to delete *Lpar5* expression. Images were then taken on D0 and DScale bar = 50 µm. *Lpar1-6* mRNA expression levels in *Lpar5^f/f^* enteroids (**B**) and *Lpar5^f/f^;Villi-Cre* enteroids (**C**) were determined and normalized to β-actin mRNA levels. Results representing three independent experiments with quadruplicates for each sample are shown. (**D**) *Lpar1–6* expression levels in *Lpar5^f/f^;Villi-Cre* enteroids (KO) were expressed relative to WT enteroids. (**E**) *Lpar1–6* mRNA expression in intestinal mucosal tissues from *Lpar5^f/f^;Villi-Cre* (KO) relative to WT mice is shown.

We have recently shown that inducible deletion of *Lpar5* in adult mice causes increased crypt epithelial cell death that dysregulates the intestinal epithelial renewal [17]. In contrast, constitutive deletion of *Lpar5* does not cause a gross change in the intestine although increased intestinal epithelial permeability is noted [14,18]. These differences are depicted in Figure 1A which compare the growth of intestinal epithelial cell (IEC)-derived enteroids from inducible *Lpar5* knockout (*Lpar5^iKO^*) and constitutive KO (*Lpar5^cKO^*) mice. Mouse intestinal crypt cells seeded in Matrigel in the presence of growth factors, including R-spondin, EGF and Noggin, and serum formed organoids or enteroids with crypt-like extensions as previously demonstrated (Figure 1A, *Lpar5^f/f^*) [3]. We have recently shown that *Lpar5* expression is almost completely depleted in *Lpar5^f/f^;Rosa-Cre^ERT^* or *Lpar5^f/f^;Ah-Cre* enteroids by treating them with 4OHT or β-NF, respectively [17]. Deletion of *Lpar5* resulted in IEC apoptosis and the enteroids lost their structural integrity (Figure 1A, *Lpar5^f/f^;Rosa-Cre^ERT^* or *Lpar5^f/f^;Ah-Cre*). In comparison, the growth of *Lpar5^f/f^;Villin-Cre* enteroids that constitutively lack *Lpar5* expression in IECs [18] was indistinguishable from wild-type (WT; *Lpar5^f/f^*) enteroids. This data correlates with what was shown in vivo in the intestinal epithelium of *Lpar5^f/f^;Villin-Cre* mice [18]. Of note, we have shown previously that 4OHT or β-NF alone has no effect on the growth of WT enteroids [17].

In light of both the striking differences between these enteroids and previous studies demonstrating overlaps in signaling by different LPARs, we hypothesized that one or more LPARs might compensate for the absence of *Lpar5* in *Lpar5^f/f^;Villin-Cre* enteroids, maintaining the survival of IECs. To examine this, *Lpar* mRNA expression levels were determined in *Lpar5^f/f^* and *Lpar5^f/f^;Villin-Cre* enteroids. Since the annealing and polymerization efficacies of the primer set for each *Lpar* are not normalized, we quantified *Lpar* mRNA expression levels relative to β-actin mRNA expression levels. Consistent with our previous studies, *Lpar1*, *Lpar5*, and *Lpar6* mRNA were more abundantly expressed in WT enteroids compared to *Lpar2*, *Lpar3*, and *Lpar4* mRNA (Figure 1B). In comparison, *Lpar2* mRNA expression was markedly elevated in *Lpar5^f/f^;Villin-Cre* enteroids (Figure 1C). In addition, *Lpar3* mRNA expression was tripled in *Lpar5^f/f^;Villin-Cre* enteroids compared with WT enteroids (Figure 1D). Expression levels of *Lpar1*, *Lpar4*, and *Lpar6* were not significantly changed. Similarly, comparing mRNA levels in mouse intestinal lysates corroborated increased *Lpar2* and *Lpar3* mRNA expression in *Lpar5^f/f^;Villin-Cre* mice compared with WT mice (Figure 1E).

**Figure 2:**
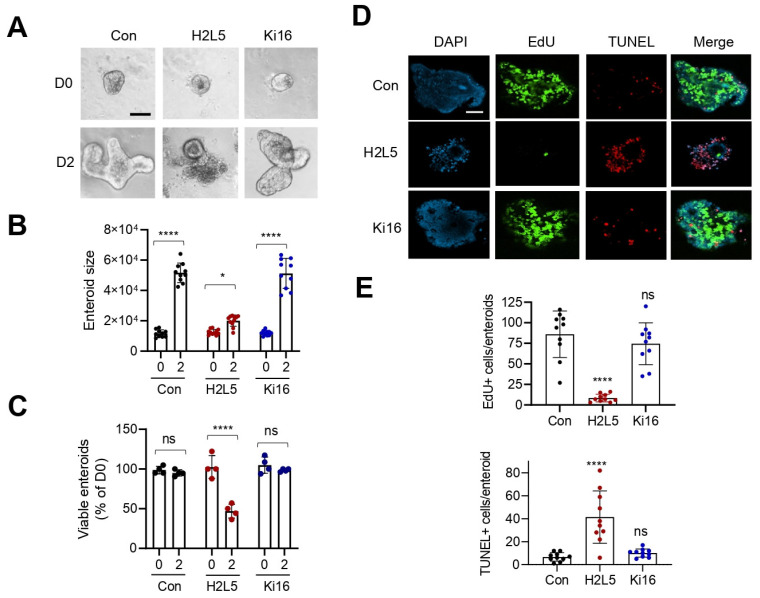
Inhibition of *LPA_2_* in *Lpar5^f/f^;Villi-Cre* enteroids results in reduced proliferation and increased apoptosis of IECs. (**A**) *Lpar5^f/f^;Villi-Cre* enteroids were treated with 10 µM LPA_2_ inhibitor H2L5186303 (H2L5) or LPA_1_/LPA_3_ inhibitor Ki16425 (Ki16) on day 0 (D0). Representative images taken on D0 and D2 are shown. *n* = Bar = 50 µm. (**B**) The growth of enteroids was quantified by determining the surface area of enteroids using ImageJ. All data are presented as mean ± SD. * *p* < 0.05, **** *p* < 0.0001 by two-way ANOVA with Tukey’s multiple comparison test. *n* = 10. (**C**) Number of viable enteroids counted on D0 and D2 and expressed as percentage on D0. *n* = 4 wells per condition. **** *p* < 0.0001, ns = not significant. (**D**) *Lpar5^f/f^;Villin-Cre* enteroids were treated with H2L5 or Ki. Representative IF images of DAPI (blue), EdU (green), and TUNEL (red) taken on D2 are shown. Bar = 50 µm. (**E**) Numbers of Edu+ (upper panel) and TUNEL+ (lower panel) cells per enteroid were quantified. *n* = 10. **** *p* < 0.0001 and ns = not significant compared with control (Con) by one-way ANOVA with Tukey’s multiple comparison test.

Given the increased *Lpar2* and *Lpar3* mRNA expression in *Lpar5^f/f^;Villin-Cre* enteroids, we next determined whether LPA_2_ or LPA_3_ contributes to the growth of *Lpar5^f/f^;Villin-Cre* enteroids. Inhibition of LPA_2_ by using LPA_2_ antagonist H2L5186303 blocked enteroid growth (Figure 2A,B) and compromised their survival (Figure 2C) [19,20]. By contrast, Ki16425, which inhibits both LPA_1_ and LPA_3_ [21], did not have a significant effect on enteroid growth or survival (Figure 2A–C). These results were reinforced by confocal immunofluorescence (IF) microscopic analysis, which showed that inhibition of LPA_2_ decreased cell proliferation (EdU), while increasing apoptosis assessed by TUNEL staining (Figure 2D). Despite increased *Lpar3* mRNA expression levels, LPA_1_/LPA_3_ antagonist Ki16425 did not alter the extent of cell proliferation or apoptosis. These results indicated that LPA_2_ compensates for the loss of LPA_5_ function to promote IEC proliferation and prevent apoptosis.

**Figure 3:**
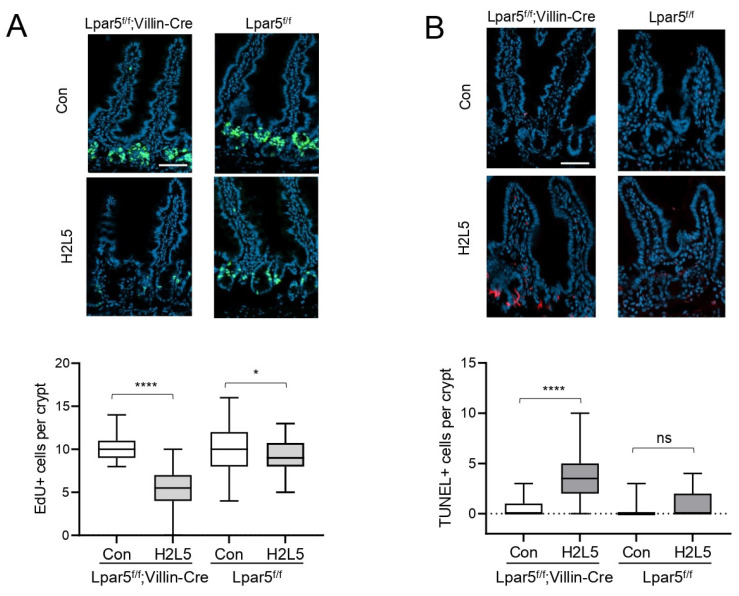
LPA_2_ inhibitor significantly alters IEC proliferation and survival in *Lpar5^f/f^;Villin-Cre* mouse intestine. Representative images of EdU (green) (**A**) and TUNEL (red) (**B**) fluorescence staining in the intestinal sections of *Lpar5^f/f^* and *Lpar5^f/f^;Villin-Cre* mice treated with DMSO (Con) or H2L5186303 (H2L5). Bar = 100 µm. Quantification of EdU+ cells per crypt and TUNEL+ per crypt are shown below. *n* = 60. * *p* < 0.05, **** *p* < 0.0001, ns = not significant by unpaired, two-tailed *t*-test.

To confirm the role of LPA_2_ on IEC proliferation and survival in the absence of LPA_5_, we administered LPA_2_ inhibitor H2L5186303 to *Lpar5^f/f^;Villin-Cre* mice at 10 mg/kg concentrations by intraperitoneal injection every other day and mice were euthanized on day Similar to the findings in enteroids, inhibition of LPA_2_ significantly decreased the number of proliferating cells in *Lpar5^f/f^;Villin-Cre* intestinal crypts while increasing apoptosis (Figure 3A,B). Interestingly, H2L5186303 led to a small but statistically significant decrease in the number of proliferating cells in control *Lpar5^f/f^* mouse crypts, suggesting that LPA_2_ may play a small role in the regulation of IEC proliferation under basal conditions.

**Figure 4:**
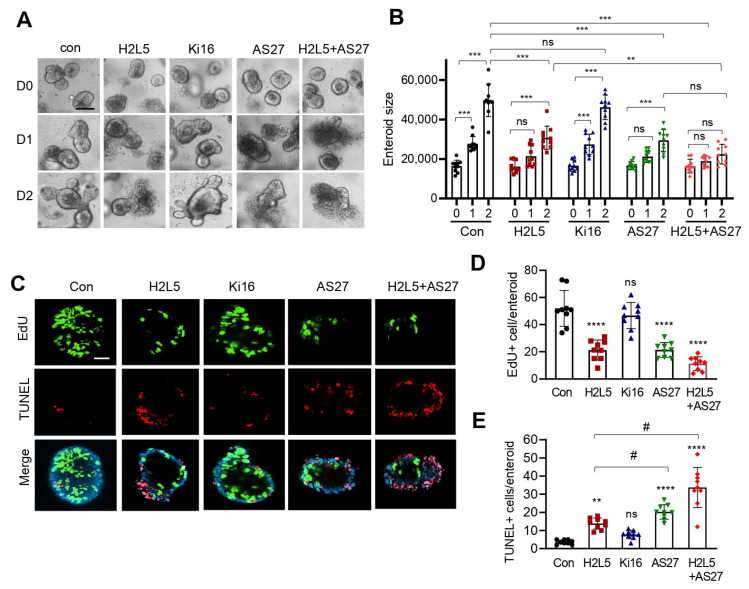
The growth of WT enteroids is suppressed by inhibition of LPA_2_ and LPA_5_. (**A**) Representative images of *Lpar5^f/f^* enteroids treated with LPA_2_ inhibitor H2L5186303 (H2L5), LPA_1_/LPA_3_ inhibitor Ki16425 (Ki16), LPA_5_ inhibitor AS2717638 (AS27), or H2L5+AS27 are shown. Data are representative of 3 experiments. Scale bar = 50 µm. (**B**) The growth of *Lpar5^f/f^* enteroids was quantified by determining the area of enteroids using ImageJ. All data are presented as mean ± SD. *n* = 10. ** *p* < 0.01, *** *p* < 0.001, ns = not significant by two-way ANOVA with Tukey’s multiple comparison test. (**C**) Representative images of EdU and TUNEL staining in *Lpar5^f/f^* enteroids treated with inhibitors are shown. Representative of three independent experiments. Bar = 50 µm. Quantification of EdU+ (**D**) and TUNEL+ **** *p* < 0.0001, ns = not significant compared to Con. (**E**) enteroid. *n* = 10. ** *p* < 0.01, **** *p* < 0.0001, ns = not significant compared to Con. ^#^ *p* < 0.0001.

To ascertain the role of LPA_2_ in promoting IEC proliferation and survival, we subjected WT enteroids to LPA_2_ inhibitor H2L5186303. Inhibition of LPA_2_ attenuated the growth of enteroids along with changes in cell proliferation and apoptosis (Figure 4A–E, H2L5 vs. Con), corroborating the in vivo findings. Consistent with our recent study that conditional deletion of *Lpar5* or LPA_5_ antagonist TC LPA5 4 blocks the growth of WT enteroids [17], LPA_5_ antagonist AS2717638 suppressed the growth of WT enteroids (Figure 4A,B, AS27 vs. Con) [22]. The effects of LPA_2_ and LPA_5_ antagonists were corroborated by IF staining of cell proliferation and apoptosis (Figure 4C–E). Simultaneous antagonism of LPA_2_ and LPA_5_ further suppressed enteroid growth compared to LPA_2_ inhibition alone (Figure 4B, H2L5+AS27 vs. H2L5), but no statistical difference was observed between enteroids treated with AS2717638 alone and those co-treated with H2L5186303+AS2717638.

**Figure 5:**
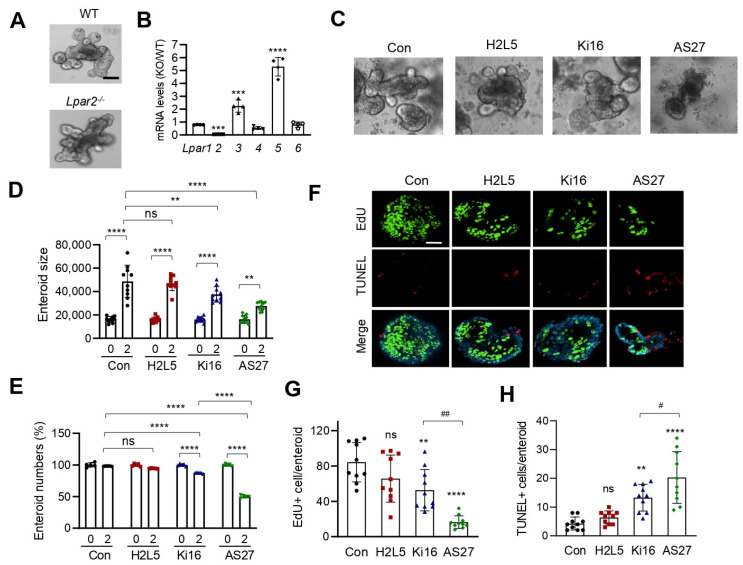
Increased *Lpar5* compensates for the loss of *Lpar2*. (**A**) Representative images of enteroids derived from WT and *Lpar2^−/−^* mice are shown. Bar = 50 µm. (**B**) Relative expression levels of *Lpar1-6* expression in *Lpar2^−/−^* compared with WT enteroids. *** *p* < 0.001, *** *p* < 0.0001 compared to WT. *n* = 4. (**C**) Representative images of *Lpar2^−/−^* enteroids treated with inhibitors are shown. (**D**) The growth of enteroids in the presence of LPAR inhibitors was quantified by determining the average surface area of enteroids using ImageJ (version 1.8, National Institutes of Health, Bethesda, MD, USA). ** *p* < 0.01, **** *p* < 0.0001, ns = not significant by one-way ANOVA with Tukey’s multiple comparison test. (**E**) The number of viable enteroids were counted on D0 and D2 and presented as % of D0. **** *p* < 0.0001, ns = not significant by two-way ANOVA with Tukey’s multiple comparison test. (**F**) Representative images of EdU (green) and TUNEL (red) staining of *Lpar2^−/−^* enteroids are shown. DAPI = blue. Bar = 50 µm. Quantification of EdU+ (**G**) and TUNEL+ cell numbers ** *p* < 0.01, **** *p* < 0.0001, ns = not significant compared to control. ^##^ *p* < 0.01 (**H**) per enteroid. *n* = Representative of three independent experiments. ** *p* < 0.01, **** *p* < 0.0001, and ns = not significant compared to control. ^#^ *p* < 0.05.

Similar to *Lpar5^cKO^* mice, constitutive knockout of *Lpar2* (*Lpar2^−/−^*) does not cause apparent phenotypic abnormality in mice and the intestinal tract appears normal under basal conditions [10,23]. Consistently, the growth of enteroids derived from *Lpar2^−/−^* mice was virtually indistinguishable from WT enteroids (Figure 5A). Hence, we investigated whether a similar compensatory mechanism prevails in the *Lpar2^−/−^* mouse intestine. In particular, given that LPA_2_ can compensate for the absence of LPA_5_, we questioned whether *Lpar5* expression is elevated in *Lpar2^−/−^* IECs. Analysis of *Lpar* mRNA expression by RT-PCR revealed elevated expression levels of *Lpar3* and *Lpar5* mRNA in *Lpar2^−/−^* enteroids compared with WT enteroids (Figure 5B). To assess whether LPA_3_ or LPA_5_ is functionally important, *Lpar2^−/−^* enteroids were cultured in the presence of LPA_1_/LPA_3_ inhibitor Ki16425 or LPA_5_ inhibitor AS2717638. We found that AS2717638 significantly inhibited the growth of *Lpar2^−/−^* enteroids (Figure 5C,D, Con vs. AS27), but Ki16425 also exerted a small but statistically significant effect on *Lpar2^−/−^* enteroids compared with controls (Figure 5D, Con vs. Ki16). As expected, LPA_2_ antagonist H2L5186303, which was used as a control, had no effect (Con vs. H2L5). These results were corroborated by IF microscopic analysis of cell proliferation (Figure 5E, EdU, and Figure 5F) and apoptosis (Figure 5E, TUNEL, and Figure 5G). AS2717638 consistently imposed a greater effect on cell proliferation and apoptosis compared with Ki16425, indicating a more dominant role of LPA_5_ in promoting *Lpar2^−/−^* intestinal crypt proliferation.

**Figure 6:**
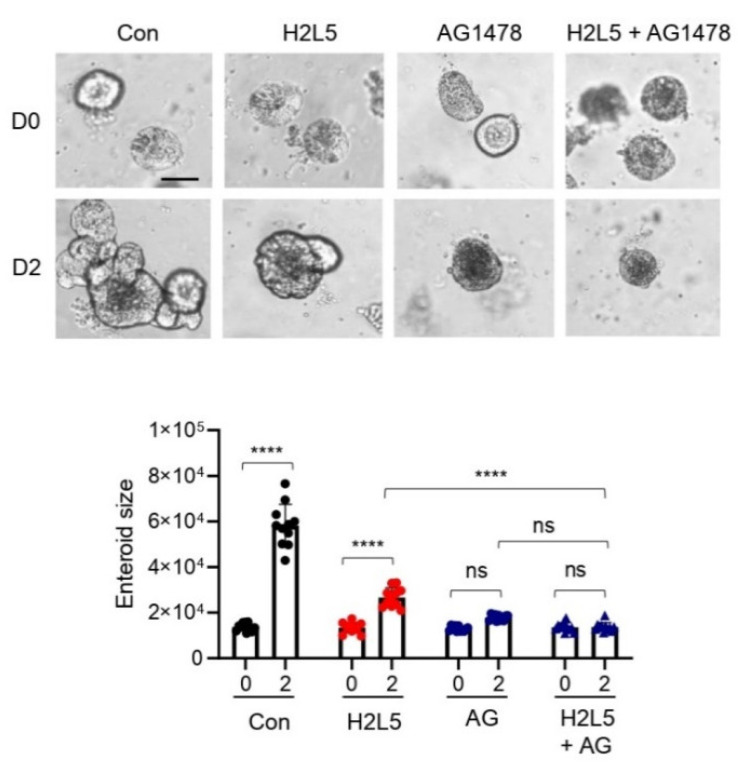
Inhibition of EGFR prevents *Lpar5^cKO^* enteroid growth. *Lpar5^cKO^* enteroids were cultured in the presence of LPA_2_ inhibitor H2L5186303 (H2L5), or EGFR inhibitor AG1478 (AG). Representative images of enteroids on D0 and D2 (upper panel) and average surface area of enteroids (lower panel) are shown. Bar = 50 μm. **** *p* < 0.0001, and ns = not significant by two-way ANOVA with Tukey’s multiple comparison test.

**Figure 7:**
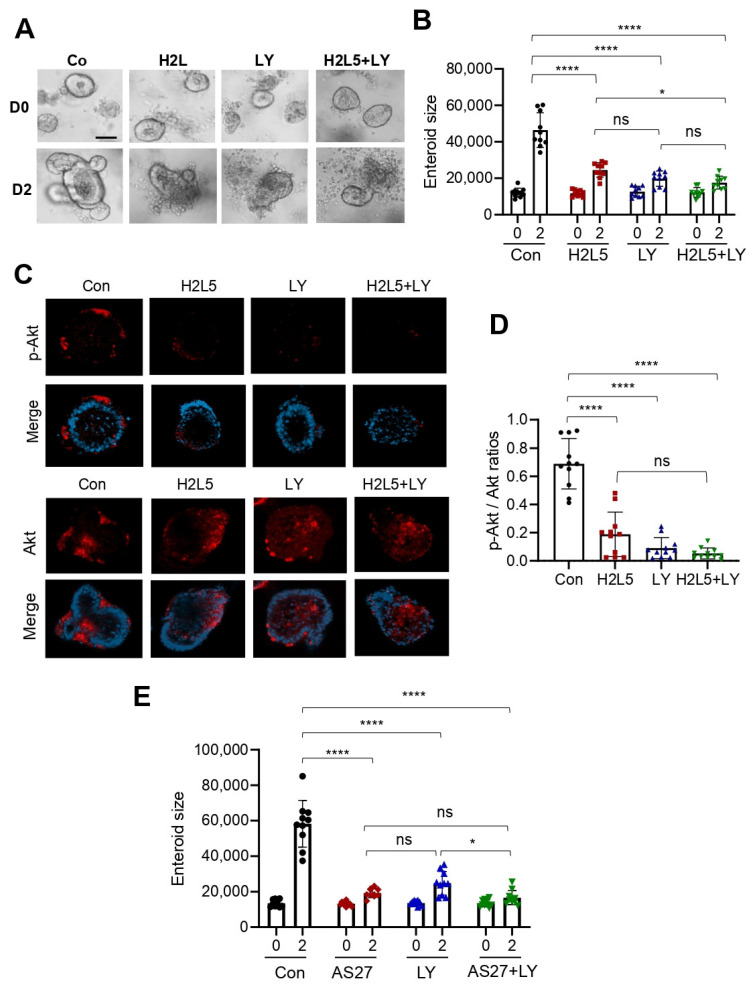
LPA_2_ promotes the growth of *Lpar5^cKO^* enteroids via activation of the PI3K-Akt pathway. (**A**) *Lpar5^cKO^* (*Lpar5^f/f^;Villin-Cre*) enteroids were cultured in the presence of LPA_2_ inhibitor H2L5186303 (H2L5), PI3K inhibitor LY294002 (LY), or both. Representative images of enteroids taken on D0 and D2 are shown. Bar = 50 μm. (**B**) Growth of enteroids was quantified by determining the average surface area of enteroids. * *p* < 0.05, **** *p* < 0.0001, and ns = not significant by two-way ANOVA with Tukey’s multiple comparison test. (**C**) Representative immunofluorescence images for p-Akt (upper panels) and Akt (lower panels) are shown. DAPI = blue. Images are representative of three independent experiments. Bar = 50 µm. (**D**) Ratios of p-Akt to total Akt fluorescence intensity (mean ± SD) are shown. *n* = 10. **** *p* < 0.0001 and ns = not significant by one-way ANOVA with Tukey’s multiple comparison test. (**E**) Growth of *Lpar2^−/−^* enteroids in the presence of LPA_5_ inhibitor AS2717638 (AS27), PI3K inhibitor LY294002 (LY), or both were determined and average surface areas of enteroids (mean ± SD) under each condition are shown. * *p* < 0.05, **** *p* < 0.0001, and ns = not significant by two-way ANOVA with Tukey’s multiple comparison test.

We next investigated the mechanism of LPA_2_-dependent growth of *Lpar5^cKO^* (*Lpar5^f/f^;Villin-Cre*) enteroids. Previous studies have shown that LPA_2_ facilitates human colon Caco-2 cell survival via activation of MAPK [24,25]. Moreover, a recent study has shown that LPA can substitute EGF to support the growth of mouse enteroids [26]. However, the stem cell culture media contain EGF which should activate EGF receptors independent of the *Lpar* expression status. This assumption was supported by the observation that inhibition of EGFR by AG1478 prevented the growth of *Lpar5^cKO^* enteroids (Figure 6). In addition to the MAPK pathway, LPA activates the PI3K-Akt pathway, which mediates proliferative signals in the intestine [25,27,28]. Therefore, we investigated the role of the PI3K-Akt pathway in sustaining proliferation and survival of *Lpar5^cKO^* IECs. We asserted the importance of PI3K in IEC proliferation by demonstrating the inhibitory effect of PI3K inhibitor LY294002 and LPA_2_ antagonist H2L5186303 similarly blocked the growth of *Lpar5^cKO^* enteroids (Figure 7A,B). Confocal IF images show the presence of phosphorylated Akt (p-Akt) in the crypt-like domains of *Lpar5^cKO^* enteroids, which was suppressed by PI3K inhibition (Figure 7C,D, LY). Importantly, a similar decrease in p-Akt levels was observed in enteroids treated with LPA_2_ antagonist H2L5186303 (Figure 7C, H2L5). Co-treatment of the enteroids with H2L5186303 and LY294002 did not yield an additive effect on enteroid growth (Figure 7A) or p-Akt (Figure 7C,D) compared to enteroids treated with either inhibitor. Taken together, these data suggest that LPA_2_ regulates the PI3K-Akt pathway, which is critical for the growth of *Lpar5^cKO^* enteroids. We next questioned whether LPA_5_-mediated effect in *Lpar2^−/−^* enteroids is also dependent on the PI3K-Akt pathway. To address this question, *Lpar2^−/−^* enteroids were cultured in the presence of LPA_5_ antagonist AS2717638, PI3K inhibitor LY294002, or both together. The growth of *Lpar2^−/−^* enteroids was halted by AS2717638, as we demonstrated in Figure Importantly, inhibition of PI3K by LY294002 effectively obliterated growth of *Lpar2^−/−^* enteroids (Figure 7E), demonstrating that the PI3K-Akt pathway is involved in the compensatory rescue of *Lpar2^−/−^* enteroids.

## 4. Discussion

The relative absence of phenotypic aberration by germline LPAR deletion suggests that either LPAR functions do not significantly contribute to the fetal development or other cellular processes compensate for the absence of the LPAR. The possibility of compensatory adaptation by another LPAR(s) is probable based on the significant overlap in the cellular signaling pathways and functions of LPARs. This study is aimed at exploring the presence of a possible compensatory mechanism in *Lpar5^cKO^* mice. We show that upregulation of LPA_2_ compensates for the loss of LPA_5_ function to maintain epithelial cell proliferation in the intestine. Our study demonstrates that LPA_2_-mediated compensatory effect is dependent on the PI3K-Akt pathway.

To identify the LPA receptor potentially compensating for the absence of LPA_5_, we utilized ex vivo cultures of intestinal epithelial stem cells from mice. The intestinal stem cells expressing LGR5 form crypt-like structures or enteroids in laminin-rich Matrigel supplemented with a cocktail of growth factors, including Noggin, R-spondin, and EGF [1]. Recently, we have demonstrated that inducible deletion of *Lpar5* compromises the intestinal crypt regeneration in vivo and impedes the formation of 3-D intestinal enteroids in vitro, demonstrating the autonomous role of LPA_5_ in the self-renewal of the intestinal epithelium [17]. In contrast, the intestinal mucosal architecture of *Lpar5^cKO^* mice appeared normal and the growth patterns of *Lpar5^cKO^* and WT enteroids were alike, suggesting a compensatory rescue of the *Lpar5* deficiency. Comparison of *Lpar* mRNA levels showed increased expression levels of *Lpar2* and *Lpar3* in *Lpar5^cKO^* enteroids, and pharmacological inhibition of LPA_2_ prevented IEC proliferation and survival. Although *Lpar3* mRNA expression was elevated in *Lpar5^cKO^* mouse enteroids, LPA_1_/LPA_3_ antagonist Ki16425 did not have a significant effect on enteroids. These results are corroborated by a recent study that Ki16425 did not have a significant effect on WT enteroids [26]. However, we cannot rule out the possibility of ineffective delivery of KiMoreover, a concern over the acute pharmacological treatment vs. the long-term effects of gene KO could not be addressed in the current study. The role of LPA_2_ in protection of IECs has been demonstrated by previous studies where activation of LPA_2_ provides protection against radiation-induced intestinal damage [29,30,31]. The presence of *Lpar2* in ISCs is not clear but a comparison of *Lpar2* mRNA along the crypt–villus axis of the mouse intestine has shown an increased abundance of *Lpar2* mRNA in the crypts compared to villi [32]. The expression of *Lpar2* in the crypts correlates with its function in epithelial regeneration. Moreover, pretreating mouse enteroids with a LPA_2_ agonist was shown to protect LGR5^+^ ISCs from irradiation [33]. Our current study confirmed the effect of LPA_2_ inhibition in vivo where a significant increase in IEC death along with decreased IEC proliferation in the intestinal crypts of *Lpar5^f/f^;Villin-Cre* mice were observed. Surprisingly, a small decrease in IEC proliferation by LPA_2_ antagonist H2L5226501 was noted in control *Lpar5^f/f^* mice, which is contrary to our previous study that no basal change in IEC proliferation was observed in *Lpar2^−/−^* mice [23]. However, we cannot rule out the possibility that the effect of H2L5226501 is caused by a lack of specificity for LPAA mouse strain with inducible *Lpar2* KO is needed to fully explore the role of LPA_2_ in epithelial regeneration in the intestine.

*Lpar1* mRNA is highly expressed in IECs, and we have observed reduced IEC proliferation in *Lpar1^−/−^* mice [34,35]. However, *Lpar1* expression was not altered in *Lpar5^cKO^* enteroids compared with WT controls, and LPA_1_/LPA_3_ antagonist Ki16425 did not have a significant effect on enteroids. The difference in IEC proliferation between *Lpar1^−/−^* mice and Ki16425 treatment of enteroids can be attributed to a probable compensatory adaptation in *Lpar1^−/−^* mice. Similarly, previous studies comparing genetic deletion of *Lpar1* and pharmacological blockage of LPA_1_ function by Ki16425 on mouse behavior and brain functions found that the antagonist mimicked some, but not all, of the effects of *Lpar1* deletion [36,37].

Because LPA_2_ compensates for the absence of LPA_5_, we questioned whether *Lpar2^−/−^* enteroids were protected by a similar compensatory process. We found increased *Lpar5* mRNA expression in *Lpar2^−/−^* enteroids and the blockade of LPA_5_ function by AS2717638 suppressed the growth of *Lpar2^−/−^* enteroids. In addition, *Lpar3* mRNA levels were doubled in *Lpar2^−/−^* enteroids relative to WT controls and LPA_1_/LPA_3_ antagonist Ki16425 appeared to have a moderate effect on *Lpar2^−/−^* enteroid growth. The effect of Ki16425 was surprising given the lack of effect on *Lpar5^cKO^* enteroids. However, one caveat of the current study is the assessment of LPAR expression by mRNA levels which do not always correlate with protein expression. We could not reliably determine LPAR protein expression due to the lack of reliable antibodies against LPAR. Nevertheless, the results in this study collectively demonstrate that a germline loss-of-function mutation of a LPAR is likely to be compensated by another LPAR. Similar to the current study, upregulation of related genes following a gene knockout has been observed previously [38]. For example, the loss of the ribosomal *Rpl22* gene results in a subtle phenotypic change due to a compensatory increase in *Rpl22-like1* gene expression [39]. Germline *PKM2*-null mice (*Pkm2*^−/−^) are viable and fertile, despite PKM2 being the primary isoform expressed in most wild-type adult tissues, due to compensatory expression of related PKM1 [40].

The EGF signaling pathway is essential for the proliferation of IECs [41], and intestinal enteroids cannot maintain their growth without EGF [3]. The transactivation of EGFR by GPCR, including LPAR, is well-established [42,43]. A recent study demonstrated the ability of LPA to substitute EGF to promote the development and growth of intestinal enteroids [26]. Consistent with the observation that LPA promotes enteroid growth through transactivation of EGFR, inhibition of LPA_1_ impeded enteroid growth in a media containing LPA but not EGF [26]. However, defective EGFR activation cannot account for the inability of *Lpar5*-deficient enteroids to grow since EGF was present in all experiments performed in the current study. Indeed, inhibition of EGFR or MEK prevented *Lpar5*-deficient enteroid growth, demonstrating that the EGF signaling was intact. LPA, present in serum in micromolar concentrations, activates multiple signaling pathways, including MEK-ERK, PI3K-Akt, phospholipase C, and p38 MAPK pathways [44,45]. We focused on the PI3K-Akt pathway based on previous studies demonstrating the critical role of the PI3K-Akt pathway in promoting IEC proliferation [27]. We found that chemical inhibition of PI3K suppressed the growth of *Lpar5^cKO^* enteroids, demonstrating the critical role of the PI3K-Akt pathway in the stimulation of IEC proliferation. Importantly, inhibition of LPA_2_ similarly attenuated activation of Akt, and blocking LPA_2_ and PI3K functions did not have an additive effect, suggesting that LPA_2_- and PI3K-dependent signaling are vertically integrated. The role of the PI3K-Akt pathway in supporting compensatory growth was recapitulated in *Lpar2^−/−^* enteroids where inhibition of LPA_5_ or PI3K blocked the growth of *Lpar2^−/−^* enteroids.

Despite evidence associating LPAR to experimental models of colitis [16,35,46], genome-wide association studies have not linked mutations in a *Lpar* gene to disorders of the gastrointestinal tract in humans. One exception to that is the identification of a single nucleotide polymorphism in GPCR GPR35 (rs4676410) among patients with inflammatory bowel disease (IBD) [47,48]. LPA is one of several endogenous ligands of GPR35, associating aberrant LPA-mediated signaling to the pathogenesis of IBD [49]. If the compensatory responses of LPAR-deficiency exist in humans, it is possible that defects associated with in-born mutations in a *Lpar* gene could be masked partially or fully by compensatory upregulation of another LPAR. Another possibility is that the extent of penetration of variant *Lpar* allele is not deep enough to provide sufficient statistical power in polygenic, multifaceted chronic inflammatory diseases. Further studies are needed to investigate whether a variant *Lpar* allele is present in a subset of IBD patients.

Here, we demonstrated that the constitutive loss of a LPAR is compensated by another LPAR. Specifically, we showed that LPA_5_ loss of function in intestinal crypt epithelium is compensated by upregulation of LPA_2_ activity, which activates the PI3K-Akt pathway. LPAR expression varies among different cell types and tissues so that there is the possibility that the compensatory adaptation in other cells, tissues, or organs might be modulated by a different member of the LPAR family. Nonetheless, this ability to compensate loss of a LPAR through upregulation of another LPAR needs to be considered in future studies that are aimed to define receptor functions or test the efficacy of a LPAR-targeting drug using genetically engineered animal models.

## Figures and Tables

**Table 1:** Primers used for qRT-PCR.

		5′-3′
*Lpar1*	Forward	CACAGTCAGCAAGCTGGTGATG
	Reverse	TCTCCGAGTATTGGGTCCTG
*Lpar2*	Forward	TCACTGGTCAATGCAGTGGT
	Reverse	AAGGGTGGAGTCCATCAGTG
*Lpar3*	Forward	ACGGCTCCCATGAAGCTAAT
	Reverse	TTCATGACGGAGTTGAGGAG
*Lpar4*	Forward	TGCATCAGTGTGGATGGTTT
	Reverse	GAAGCCTTCAAAGCAAGTCG
*Lpar5*	Forward	GCTCCAGTGCCCTGACTATC
	Reverse	GGGAAGTGACAGGGTGAAGA
*Lpar6*	Forward	TGACTGTGAACCACAGAGCA
	Reverse	ACTTTGCAACACGGAATTGG
*ACTB*	Forward	AGCCATGTACGTAGCCATCC
	Reverse	TCTCAGCTGTGGTGGTGAAG

## Data Availability

Not applicable.
